# Cardiac troponin T and echocardiographic dimensions after repeated sprint vs. moderate intensity continuous exercise in healthy young males

**DOI:** 10.1038/srep24614

**Published:** 2016-04-19

**Authors:** Matthias Weippert, Dimitar Divchev, Paul Schmidt, Hannes Gettel, Antina Neugebauer, Kristin Behrens, Bernd Wolfarth, Klaus-Michael Braumann, Christoph A. Nienaber

**Affiliations:** 1 Institute of Sport Science, University of Rostock, Germany; 2 Institute of Exercise Physiology and Public Health, Rostock, Germany; 3 Department of Cardiology, Campus Marburg, University Clinic Giessen and Marburg, Germany; 4 Department of Sports Medicine, Humboldt-University/Charité University Medicine, Berlin, Germany; 5 Institute of Human Movement Science, University of Hamburg, Hamburg, Germany; 6 Department of Cardiology, Center for Internal Medicine, Rostock University Medicine, Rostock, Germany

## Abstract

Regular physical exercise can positively influence cardiac function; however, investigations have shown an increase of myocardial damage biomarkers after acute prolonged endurance exercises. We investigated the effect of repeated sprint vs. moderate long duration exercise on markers of myocardial necrosis, as well as cardiac dimensions and functions. Thirteen healthy males performed two different running sessions (randomized, single blinded cross-over design): 60 minutes moderate intensity continuous training (MCT, at 70% of peak heart rate (HR_peak_)) and two series of 12 × 30-second sprints with set recovery periods in-between (RST, at 90% HR_peak_). Venous blood samples for cardiac troponin T (cTnT), creatine kinase (CK) and MB isoenzyme (CK-MB) were taken 1 and 4 hours after exercise sessions. After each session electrocardiographic (ECG) and transthoracic echocardiographic (TTE) data were recorded. Results showed that all variables - average heart rate, serum lactate concentration during RST, subjective exertion and cTnT after RST - were significantly higher compared to MCT. CK and CK-MB significantly increased regardless of exercise protocol, while ECG and TTE indicated normal cardiac function. Our results provide evidence that RST contributes significantly to cTnT and CK release. This biomarker increase seems to reflect a physiological rather than a pathological phenomenon in healthy, exercising subjects.

Sport and medical studies have consistently shown an increase in cardiac biomarkers during and following prolonged endurance exercise^1^,^2^,^3^. Besides exercise duration, its intensity is also a proposed significant contributor to cardiac troponin (cTn) release^4^. However, although repeated sprints (RS) are common in team sports and significantly contribute to athletic performance and aerobic fitness in other types of sport, their effect on the release of biochemical markers of myocardial damage has not been addressed yet. RS training (RST) is characterized by very short exercise bouts (maximal 30 seconds) of vigorous intensity and hence differs from long duration moderate intensity continuous training (MCT). While increases of cardiac troponin I (cTnI) were reported after high intensity continuous exercise (30 minutes)^5^, there was no evidence of a significant release of troponin cTn after high intensity intermittent exercise of short duration in adults^6^,^7^. Based on these findings, increased cTn after RS activities would suspect cardiac damage. However, little is known about the biomarker kinetics in relation to exercise intensities and durations as they frequently occur in sports such as competitive endurance training, handball, hockey, basketball, or other types of physical activity^8^,^9^,^10^. Further, while short duration intense exercise may impact on the release of cTn in adolescents^11^,^12^ this does not seem to apply to adults^13^.

Training regimens including short duration high intensity exercises are known to be more efficient to improve peak oxygen uptake (VO_2peak_), performance and running economy compared to moderate intensity high volume training and training at anaerobic threshold^10^,^14^. An improvement of VO_2peak_ in high intensity groups was found to correlate with an increase in stroke volume^14^. Although RST has been commonly applied in endurance training, in team sports, and even in cardiac rehabilitation studies, its effect on markers of cardiac stress has not been fully investigated yet. In light of conflicting results regarding the effect of exercise intensity on morbidity and mortality^15^,^16^,^17^,^18^, it appears to be of importance to elucidate the relationship between exercise “dose”, cardiac biomarker release and cardiac function. In addition, better understanding exercise-induced releases of cardiac biomarkers may have a valuable diagnostic impact if individuals are admitted to medical care after vigorous exercise.

The aim of this study was to test the impact of RST on biomarkers of acute myocardial necrosis by comparing results to those of traditional MCT. Myocardial dimensions and functions were assessed by transthoracal echocardiograpic (TTE) and electrocardiographic (ECG) investigations following each exercise session. We hypothesized, that despite its short total duration, RST training would significantly increase cTnT and CK-MB, compared to MCT of longer duration. Hypothesis confirming results would elucidate the prevalence of an exercise-induced release of myocardial necrosis biochemical markers complemented by additional information on the dose – response relationship between exercise, biomarker release and myocardial adaptations to high intensity training.

## Methods

This study was performed in compliance with the Declaration of Helsinki and approved by the local ethics committee at the University of Rostock (approval number: A 2014-0066).

Thirteen healthy and aerobically trained male volunteers (age: 26.2 ± 2.9 years, body height: 182.4 ± 6.4 cm, body weight: 80.6 ± 7.0 kg, VO_2_max: 52.2 ± 3.7 ml*min^−1^*kg^−1^)^19^ gave their written informed consent to participate in this study. Inclusion criteria were predefined as follows: no known cardiovascular diseases or musculoskeletal disorders/injuries, and a current medical clearance for exercise participation. Participants were asked to refrain from alcohol one day before experiments and from any exhaustive exercise at least two days prior to testing. Nicotine, alcohol and caffeine were not allowed on all experimental days. Prior to the first exercise session, all participants underwent a careful cardiac examination assuring physical eligibility for this study. Resting 12-lead ECGs were analyzed manually by the study physician applying the Seattle criteria for normal electrocardiographic findings^20^. Further, we assessed TTE indices to evaluate cardiac function according to current guidelines^17^,^20^,^21^. No pathological findings were detected for ECG or TTE indices.

Individual peak heart rate (HR_peak_) was used to control the individual workload during the different training sessions. To determine individual HR_peak_, participants were instructed to speed up as fast as possible during the last 30 seconds of a maximal all out 12-minute run one week prior to the first training session.

The study was designed as a single-blinded randomized cross-over trial, with participants performing two different running sessions on a 400-meter track. On the first training day, subjects were randomly assigned to start with either a RST session including 5 minutes warm-up, followed by two series of 12 × 30-second sprints with 15 seconds recovery between the sprints and 3 minutes between the interval series^22^, or a 60-minute MCT. After a “washout”-period of at least five days the respective second training session was applied. ECG and TTE were screened for pathological findings and functional changes. ECG was recorded 15 minutes after each exercise session using a Cardiax PC-ECG USB-Version (IMED, Budapest, Hungary). TTE (GE Vivid 7 Dimension^®^ ultrasound system, General Electric Company, Fairfield, Connecticut, USA) was performed 30 minutes after the cessation of RST and MCT, to assess left and right diastolic and systolic function according to current guidelines^17^,^20^,^21^. Left heart function was assed using ventricular end-systolic (LVESD) and end-diastolic diameter (LVEDD), left ventricular ejection fraction (LVEF), maximum trans-aortic velocity (AoV_max_), mitral inflow velocity E-wave (E), mitral anular E-wave (E′) in tissue Doppler imaging (TDI) and Fractional shortening (FS). The E/E′ relationship and pulmonary capillary wedge pressure (PCWP) were calculated using the Nagueh-Formula: PCWP = 1.9 + (1.24 E/E′). Right heart function was assessed by ventricular end-diastolic diameter (RVEDD), maximum trans-pulmonal venous velocity (PVVmax), tricuspid annular plane systolic excursion (TAPSE), tricuspid annular systolic velocity (TASV) as well as mean and diastolic pulmonary regurgitation gradient (PA_mean_ and PA_dia_) derived from pulmonary regurgitation.

Venous blood samples for cardiac troponin T (cTnT), creatine kinase (CK) and MB isoenzyme of creatine kinase (CK-MB) were taken before (PRE), as well as 1 hour (POST +1) and 4 hours (POST +4) following cessation of exercise. Cardiac troponin T (cTnT) was detected using a 5^th^ generation highly sensitive assay (Elecsys Troponin T hs assay, Roche)^23^. Its limit of blank is 3 ng/L, its precision is <10% CV (at 13 ng/L) and the upper reference limit (99^th^ percentile) for men is 14.5 ng/L^24^,^25^. CK activity was measured using CK-Nac (BECKMAN COULTER) providing a range from 10–2000 U/L and high total precision (CV: <10%). CK-MB was measured using the CKMB UDR reagent (SENTINEL DIAGNOSTICS, range: 5–2300 U/L, high total precision: <5% CV). Measurement of all biochemical markers was carried out according to the IFCC-Guidelines^26^. TTE investigators and laboratory staff were blinded to what type of exercise session had been accomplished.

We measured HR during each exercise session with a Polar® heart rate monitor (Polar Electro, Kempele, Finland)^27^ and we used relative HR (percentage of individual peak HR, %HR_peak_) to assess individual exercise intensity. To determine blood lactate concentration we drew a capillary blood sample from the participant’s earlobe (LactateScout, Senslab, Leipzig, Germany) just 1 minute after exercise cessation.

So far, cross-over high-intensity intermittent exercise studies have lacked relevant data for average exercise-induced 5^th^ generation cTnT elevations. Thus, a priori sample size calculation was based on an average value of 6 ng/L after MCT; that is roughly the 50^th^ percentile of a reference population for this assay. Based on research findings in adolescent basketball players^11^ we estimated an increase of cTnT above the cut-off (99^th^ percentile, 14.5 ng/L) after RST for half of the potential sample, with the other half showing cTnT activity comparable to the MCT session (6 ng/L). Based on these assumptions, sample size calculations with an effect size of 1.3 and α-error probability of 0.05 require 10 participants to detect significant effects (G*Power 3.1.9.2, University of Kiel). Taking into account a possible drop-out rate of one third, we considered a sample size of thirteen persons as appropriate to study effects of exercise regimen on cTnT-release.

We tested the effect of exercise protocol on exercise HR, blood lactate concentration and RPE by Wilcoxon’s rank sum test for matched pairs (IBM SPSS Statistics 20.0). A two factor ANOVA for repeated measures was applied (IBM SPSS Statistics 20.0) to test the effects of time (PRE, POST +1, POST +4) and exercise protocol (RST vs. MCT) on biomarker release. If data violated the statistical assumption of sphericity, Greenhouse-Geisser corrected p-values and respective degrees of freedom were reported. For post-hoc pair wise comparisons significance levels were adjusted using Bonferroni’s correction. The effect of exercise protocol on TTE indices, while controlling for a confounding effect of HR on TTE-measurements, was calculated with a two factor analysis of covariance for repeated measures (ANCOVA, IBM SPSS Statistics 20.0).

Spearman’s correlation coefficient was calculated to assess the relationship between exercise intensity and biomarker release as well as between normalized cardiac dimensions and biomarker release after RST.

## Results

The average HR_peak_, calculated from the measurements at the end of each 12-minute run, was 190.4 ± 6.4 bpm (range: 181–201 bpm). Table 1 shows absolute and relative HR during exercise sessions; the average HR during MCT corresponds to “moderate intensity”, while HR during RST corresponds to “hard or vigorous” according to the ACSM guidelines^9^. There was a significant difference between the two conditions for HR, lactate concentration and RPE (Table 1).

**Figure 1:**
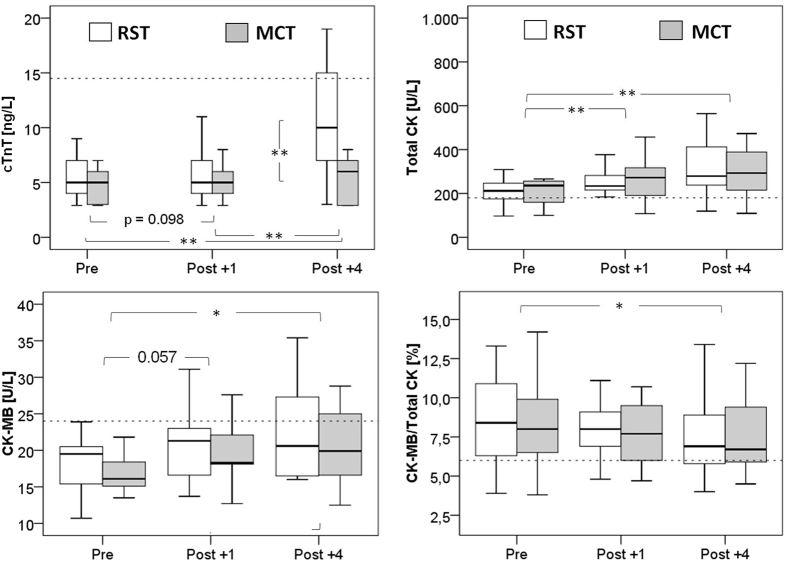
Biomarker concentration before (PRE), 1 (POST + 1) and 4 hours (POST + 4) after cessation of the different exercise protocols; ^*^p < 0.05, ^*^^*^p < 0.001, dotted line = reference limit; MCT, moderate intensity continuous training; RST, repeated sprint interval training; cTnT, cardiac troponin T; CK, creatine kinase; CK-MB, CK muscle-brain isoform; %CK-MB, ratio of CK-MB: total CK in %, N = 13.

Statistical analysis revealed a strong effect on cTnT by means of measurement time (F(1.2,14.5) = 18.43, p < 0.001, η^2^ = 0.606), exercise protocol (F(1,12) = 17.483, p < 0.01, η^2^ = 0.593) as well as their interaction (F(1.2,13.1) = 19.684, p < 0.001, η^2^ = 0.621). Figure 1 shows PRE, POST +1 and POST +4 biomarker concentrations and significance levels for the post hoc pair wise comparisons. CTnT significantly increased 4 hours after exercise; a statistical trend was evident for POST +1. In five out of the thirteen participants, cTnT exceeded the clinical limit of normal.

Time of measurement also had a significant effect on total CK (F(1.1,13.5) = 13.176, p < 0.01, η^2^ = 0.523), CK-MB (F(2,24) = 6.287, p < 0.01, η^2^ = 0.344) and on the percentage of CK-MB to total CK (F(2,24) = 4.097, p < 0.05, η^2^ = 0.255). On the other hand, there was no significant impact on total CK and CK-MB by either type of exercise or by the interaction of measurement time and training protocol. However, total CK significantly increased from PRE to POST +1; while CK increase from POST +1 to POST +4 was not significant. CK-MB progressively increased from PRE to POST +4. In more detail, CK-MB activity was above the reference limit of 24 U/L for one of the participants at RST PRE, for three participants at RST POST +1, for six at RST POST +4, for two at MCT PRE, for three at MCT POST +1, and for four participants at MCT POST +4. Percent CK-MB declined 4 hours post exercise. The percentage of CK-MB to total CK was elevated above the 6% limit in twelve out of thirteen participants at RST PRE and MCT PRE, respectively. One hour after exercise %CK-MB was above 6% in nine participants after RST and in eleven participants after MCT. At POST +4 the %CK-MB exceeded the reference limit in eight and in ten participants, respectively.

Furthermore, there was a significant correlation between cTnT POST +4 release and exercise blood lactate concentration (Spearman’s r = 0.542, p < 0.01), RPE (Spearman’s r = 0.617, p < 0.001) and %HR_peak_ (Spearman’s r = 0.692, p < 0.001). While cTnT at POST +4 did not reveal any correlation, CK-MB at POST +4 showed a trend (r = 0.498, p = 0.083) and CK a moderate association with BMI-normalized LVEDD at BL (r = 0.634, p = 0.020).

TTE indices indicated normal morphology and function after both exercise protocols (Table 2). None of the participants showed impaired cardiac function according to TTE evaluation, neither after MCT, nor after RCT. While diastolic and systolic blood pressure did not differ after recovery from RST and MCT (SBP after RST: 113.6 ± 6.1 mmHg vs. SBP after MCT: 111.9 ± 5.7 mmHg, p = 0.586; DBP after RST: 72.3 ± 7.2 mmHg vs. DBP after MCT: 74.5 ± 8.1, p = 0.893), respectively, HR was still elevated during TTE (HR after RST: 89.3 ± 7.8 bpm vs. HR after MCT: 77.0 ± 11, p < 0.001). ANCOVA showed that, after correction for HR, the exercise protocol had a significant effect on PVV_max_ only, while all other differences were statistically insignificant.

## Discussion

This is the first study to demonstrate an effect of short duration RST on cardiac biomarker kinetics in healthy adults. We found that – in contrast to MCT – RST significantly increased cTnT; with five out of thirteen participants showing values above the upper reference level of 14.5 ng/L. On first sight, these findings may be controversial, since George and colleagues found no release of cTnT after high intensity short-duration intermittent exercises^6^. However, for their team sport athletes, blood samples were drawn 30 minutes and 24 hours after a rugby/ football game, respectively, while in our study we analyzed blood samples drawn 1 and 4 hours after RST. A less sensitive 3^rd^ generation cTnT method used in the referenced study might also account for the difference between those and the present findings. Further, not only timing and sensitivity of measurements, but also duration of single exercise bouts might have been shorter and recovery longer in previous studies compared to the RST in our study. For instance, cTnI release after soccer specific exercise sessions was not elevated^7^. Moreover, intermittent exercises with longer intervals (23 × 2 minutes at velocity of 90% VO_2max_ with 2 minutes at 50% VO_2max_ velocity in between) under normoxic and hypoxic conditions in adult marathon runners^28^ showed that cTn was unrelated to environmental conditions. The authors then concluded that exercise intensity seems to be a modulator. However, considering the fact that total exercise duration in the referenced study corresponded to running times of a half marathon or even beyond, such increases of cTn are known to be common^29^. Thus, based on these previous findings, the impact of exercise intensity alone on markers of myocardial damage had remained uncertain. Results of our study clearly demonstrate that – compared to exercises of longer duration and moderate intensity – short duration high intensity intermittent exercise significantly affects the release of cardiac biomarkers in a healthy adult population.

As ECG and TTE indicated normal cardiac function after both training sessions, we conclude that the release of cTnT after RST is a physiological and not a pathological phenomenon^30^,^31^. Biomarker releases might be an indicator of an effective training stimulus, necessary to promote functional and morphological adaptation to high intensity intermittent exercise^31^. Correlation analyses showed an association between cTnT release and the metabolic state of the working skeletal muscle, i.e. exercise “dose”. This indicates that peripheral anaerobic skeletal metabolism, resulting in an altered acid-balance and/or an increased production of oxidative radicals, could be a contributing factor to the loss of cardiomyocytes’ integrity^29^. Conclusions are preliminary, as we also found a significant relationship between cTnT with %HR_peak_, which stands in contrast to the findings of Li *et al*.^28^. The latter would support the view of mechanical stress on cardiomyocytes as a significant mechanism contributing to the release of cTn after exercise^29^,^31^. It has been shown in trained subjects, that stroke volume (SV) progressively increases with rising HR until maximal effort^32^,^33^. Thus, in contrast to the MCT session, RST was expected to induce SVs close to maximum and with that puts greater mechanical stress on cardiomyocytes. In essence, exercise-induced biomarker releases after RST in young active males seem to reflect a loss of cellular integrity due to repeated maximal stretch of the cardiomyocytes and/or other factors associated with high intensity exercise. Our findings indicate exercise intensity as a significant contributor to the cTnT-release during exercise durations ≤ 60 minutes. A recent study reported that a single bout of maximal exercise (30 seconds) does not significantly affect cTnT release^13^. However, our experiment demonstrates that repeated 30-second intervals of high, but not maximal, intensity with short recovery times can significantly elevate cTnT concentration. The sampling strategy in the present study, including measurements at 1 and 4 hours post exercise only, might be a limitation, as others have documented an individual time dependent cTnT release during and post exercise^29^. Hence, despite revealing a significant effect of exercise intensity on cTnT release, not all “responders” might have been detected. After all, our sampling procedure was justified by the following two arguments: First, we considered a typical critical scenario at emergency care, e. g. submission of a male endurance athlete with exercise associated syncope of unknown origin; and second, other studies revealed that 2 hours following the start of a moderate intensity exercise (marathon) all participants showed elevated cTnT values^34^.

It remains however speculative whether the release of cTnT already indicates an exercise stimulus as sufficient to induce long-term functional and structural cardiovascular adaptations. The physiological “damage” underlying the increase of cTn may further explain the superior effect of RST on cardiovascular function, compared to moderate continuous exercise evident in different samples^8^,^22^.

In regards to the other biomarkers measured in our study, we found that total CK and CK-MB significantly increased after either exercise protocol. Our study supports both: previous findings of increased CK-values in athletes, and the notion that CK-values above reference limits are the rule rather than exception in active young males or athletes even under resting conditions^23^,^35^,^36^,^37^. After RST more participants showed CK-values above the upper reference compared to after MCT. As this result appears in line with the release of cTnT after RST, a myocardial origin of the measured CK-MB after RST is not unlikely^38^. This result supports the notion of an increased myocardial stress induced by RST. However, in contrast to our hypothesis the impact of the exercise type on CK-MB was statistically not significant. CK-MB was above 6% of total CK both at rest and after exercise. This leads to the conclusion that %CK-MB has no incremental diagnostic value in active young males with suspected acute myocardial infarction. However, the time course of such (nonspecific) markers may be instrumental to improve the medical decision making process after an exercise-associated hospital submission. A typical constellation in healthy individuals after high intensity short duration exercise seems to be an increase in total CK from POST +1 to POST +4, while CK-MB remains at a stable level. In addition, there was a statistical trend (p = 0.07) for a decrease of %CK-MB from POST +1 to POST +4. This suggests that a progressive decline of %CK-MB in the first hours after training indicates an exercise associated “physiological” release of CK-MB without any relevant pathology, as confirmed by perfectly normal myocardial function.

In summary, our data support the notion that exercise intensity is an important factor to explain moderate increases in cTnT^4^,^39^. While 60 minutes of continuous endurance exercise at moderate intensity did not trigger a significant release of cTnT, high intensity intermittent exercise did. Because the release of cTnT exceeded normal limits in almost half of the studied individuals, we advise that the type of exercise should be considered whenever moderate increases of myocardial markers are noticed. A better understanding of an exercise-induced release of cTnT and CK-MB might be of diagnostic importance if an individual is admitted to medical care after exercise^5^,^29^. It remains to be investigated whether healthy subjects with a more pronounced elevation of cTnT after intermittent exercise (“responders”) might benefit more from RST in terms of SV increases (e.g. via eccentric cardiac hypertrophy) than “non-responders”. The significant correlation between normalized left-ventricular dimensions suggests that non-specific total CK might be a trigger of morphological adaptations in response to regular exercise. When considering HR during TTE as a covariate, results show that the type of training hardly affects post-exercise echocardiographic indices of myocardial function in healthy active men. Compared to baseline there was also no sign of impairment after either training session.

Admission to emergency care after strenuous exercise with associated cTnT elevations has frequently led to extensive but unnecessary clinical workup. Clinicians should be aware that exercise-induced releases of cTnT, CK and CK-MB are not only limited to result from prolonged endurance exercises, like marathon running, but may also occur after short duration intermittent activity, like repeated sprint training or game sports. We suggest that with a better understanding of the proposed exercise and seromarkers relation in both trained and sedentary individuals, some futile workup based on a false positive diagnosis can be avoided^40^.

## Additional Information

**How to cite this article**: Weippert, M. *et al*. Cardiac troponin T and echocardiographic dimensions after repeated sprint vs. moderate intensity continuous exercise in healthy young males. *Sci. Rep.* **6**, 24614; doi: 10.1038/srep24614 (2016).

## Figures and Tables

**Table 1:** Absolute and relative heart rate during different exercise sessions (N = 13).

**Session**	**RST complete session (incl. between-series recovery, warm-up and cool-down, duration: 29 minutes)**	**RST (intermittent sprint exercise only, duration: 17.5 minutes)**	**MCT (60 min continuous running)**
HR [1/min]	**152.4** ± 24.9*	**174.1** ± 9.8**	**136.3** ± 6.2
%HR_peak_	**80.1** ± 13.0*	**91.6** ± 3.4**	**71.6** ± 2.7
Blood lactate [mmol/L]	–	**5.9** ± 1.6**	**1.6** ± 0.7
RPE [Borg scale: 6–20]	–	**16.8** ± 1.7**	**11.2** ± 1.7

MCT – moderate intensity continuous training, RPE – rating of perceived exertion, RST – repeated sprint interval training, */**) significantly different from MCT, p < 0.05/ p < 0.001.

**Table 2:** Mean ± SD of TTE indices at Baseline, after RST and after MCT (significance of the difference between RST and MCT assessed by ANCOVA).

	**Baseline**	**0.5 h after RST**	**0.5 h after MCT**	***p***
	**Mean ± SD**	**Mean ± SD**	**Mean ± SD**	
LVESD [mm]	**32.6** ± 1.9	**31.9** ± 1.9	**32.9** ± 2.8	0.472
LVEDD [mm]	**53.6** **±** **2.8**	**53.8** ± 2.0	**52.7** ± 2.3	0.658
FS [%]	**37.6** ± 4.1	**40.6** ± 2.8	**37.5** ± 5.5	0.471
LVEF [%]	**64.3** **±** **9.9**	**65.3 ± 9.8**	**65.9** ± 4.0	0.647
RVEDD [mm]	**38.2** ± 2.7	**37.6** ± 2.1	**37.4** ± 2.7	0.838
AoV_max_ [cm/sec]	**178.7** ± 15.7	**176.9** ± 14.1	**176.8** ± 17.9	0.767
PVV_max_ [cm/sec]	**111.6** ± 20.3	**106.8** ± 13.0	**102.8** ± 13.7	0.007
E [cm/sec]	**83.7** ± 20.8	**70.4** ± 16.6	**85.3** ± 10.5	0.470
E′ [cm/sec]	**19.1** ± 3.8	**18.5** ± 4.1	**18.2** ± 3.5	0.624
E/E′	**4.4** ± 1.0	**3.9** ± 0.7	**4.8** ± 0.8	0.661
mPCWP [mmHg]	**7.4** ± 1.2	**6.7** ± 0.8	**7.9** ± 1.0	0.673
PA_mean_ [mmHg]	**9.6** ± 1.1	**8.8** ± 0.9	**9.9** ± 1.1	0.167
PA_dia_ [mmHg]	**7.6** ± 1.0	**6.6** ± 0.8	**5.3** ± 10.5	0.193
TAPSE [mm]	**24.8** ± 1.6	**22.3** ± 2.4	**23.7** ± 1.4	0.753
TASV [cm/sec]	**15.7** ± 2.3	**13.5** ± 2.8	**14.6** ± 1.6	0.646

E – mitral inflow velocity E-wave, E′ – mitral-anular E-wave, FS – fractional shortening, LVEDD – ventricular end-systolic diameter, LVEF – left ventricular ejection fraction, LVESD – ventricular end-diastolic diameter, MCT – moderate intensity continuous training, PA_dia_ – diastolic pulmonary regurgitation gradient, PA_mean_ – mean pulmonary regurgitation gradient, PVV_max_ – trans-pulmonal velocity, PCWP – pulmonary capillary wedge pressure, RST – repeated sprint interval training, RVEDD – right ventricular end-diastolic diameter, TTE – transthoracal echocardiography, TAPSE - tricuspid annular plane systolic excursion, TASV - tricuspid annular systolic velocity.
